# Sleep Encephalography‐Based Brain Age Index as a Potential Biomarker for Incident Cognitive Impairment in Community‐Dwelling Older Men

**DOI:** 10.1002/alz.089971

**Published:** 2025-01-09

**Authors:** Haoqi Sun, Sasha Milton, Robert J Thomas, M. Brandon Westover, Katie L Stone, Kristine Yaffe, Yue Leng

**Affiliations:** ^1^ Beth Israel Deaconess Medical Center, Boston, MA USA; ^2^ Massachusetts General Hospital, Boston, MA USA; ^3^ University of California, San Francisco, San Francisco, CA USA; ^4^ Departments of Psychiatry and Behavioral Sciences, Neurology, and Epidemiology, University of California San Francisco, San Francisco, CA USA

## Abstract

**Background:**

Individuals with dementia frequently exhibit disturbed sleep electroencephalography (EEG) microstructures, including spindles and slow oscillations. We have previously developed a sleep EEG‐based brain age index (BAI), which integrates multiple sleep EEG microstructures and has been associated with prevalent dementia in clinical populations. Here, we aim to determine if BAI is associated with incident cognitive impairment (CI) in a community population of older men, and its discrimination capability as a digital biomarker.

**Method:**

This longitudinal study included men older than 65 years who completed overnight polysomnography (PSG) between 2003 and 2005 and had incident CI followed until 2016, as part of the Osteoporotic Fractures in Men study (MrOS). BAI was based on a model trained on participants without neurological or psychiatric diseases. Incident CI was defined as self‐reported dementia, dementia medication use, Modified Mini‐Mental State Examination (3MS)<80, or a decline in 3MS from baseline to any following visit ≥1.5 standard deviations below the mean. We fit logistic regression to assess the associations between quintiles of BAI and incident CI.

**Result:**

There were 2,609 men without dementia at baseline (mean age [sd] = 76.0 [5.3] years), of whom 416 developed incident CI during a maximum follow‐up of 11 years. After adjustment for demographics, education, baseline cognition, medication use, comorbidities, and lifestyle, the highest quintile of BAI (BAI>+2.3 years) was associated with incident CI with an odds ratio of 1.66 (95% confidence interval 1.17–2.38, p=0.0051) when compared to the lowest quintile (BAI<‐6.7 years). Other quintiles were not significantly associated with incident CI. BAI showed a cross‐validated accuracy of 60%, sensitivity of 66%, specificity of 55%, and area under the receiver operative curve of 61% for incident CI. When looking at the brain age itself, the brain age performed better than chronological age in predicting incident CI, since the model with brain age achieved lower (i.e., better) Bayesian Information Criterion than another model with age, together with other covariates.

**Conclusion:**

Higher BAI was associated with incident CI in community‐dwelling older men. These results highlight the potential of BAI as a digital biomarker of future CI in older men.

